# Supplementary material for: The 6-item specific object anthropomorphism scale: a new questionnaire for children and adults
Source: PeerJ. 2025 Oct 9;13:e20153. doi: 10.7717/peerj.20153 (PMC12515426; doi:10.7717/peerj.20153)
Supplement: Supplemental Information 1 [file peerj-13-20153-s001.docx]

**Supplementary Materials**

**Expert Review**

**Supplementary Table 1** *Initial Item Pool Given to N = 23 Experts*

| Item | Mean Relevance | Mean Readability |
| --- | --- | --- |
| 1. This object can feel happy or sad. | 3.57 | 3.71 |
| 2. This object can be naughty or nice. | 3.13 | 3.43 |
| 3. This object can think. | 3.87 | 3.95 |
| 4. This object can do what it wants. | 3.30 | 3.52 |
| 5. This object has opinions. | 3.52 | 3.81 |
| 6. This object understands people. | 3.22 | 3.38 |
| 7. This object can be kind or mean. | 3.23 | 3.57 |
| 8. This object needs friends. | 3.17 | 3.62 |
| 9. This object is easy to talk to. | 2.83 | 3.48 |
| 10. This object needs a first name. | 2.87 | 3.43 |
| 11. This object knows what is happening around it. | 3.39 | 3.33 |
| 12. This object is alive. | 3.26 | 3.84 |

*Note*. Relevance and Readability were rated on 4-point Likert scales from 1 (*Completely Irrelevant/Unclear*) to 4 (*Very Relevant/Clear*).

**Supplementary Figure 1**

*Object Used in All Three Studies (Crocheted Cactus)*


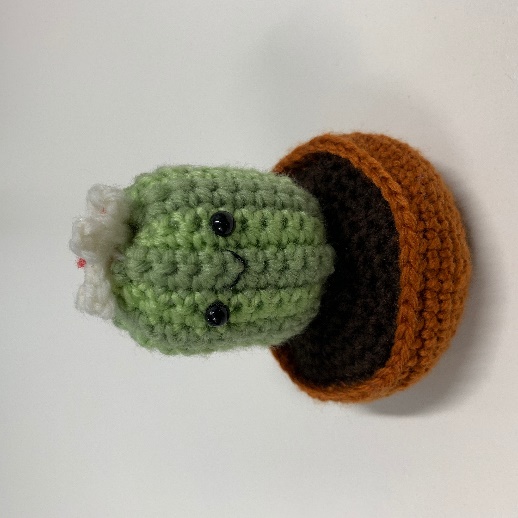


**Study 2 Results with the 6-Item SOAS**

We conducted confirmatory factor analysis with the 6-item SOAS (excluding items 1, 2, 3, 4, 6, 8, & 13) and found excellent model fit (χ^2^[9] = 7.20, *p* = .62, CFI = 1.00, TLI = 1.00, SRMR = 0.02, RMSEA < .001 [90%CI = < .001, .05]). See Supplementary Table 2 for standardised factor loadings.

The 6-item SOAS also demonstrated excellent internal reliability (ω = .90) and good test-retest reliability (ICC = .74) when the measure was completed on average 15 days later. The correlations between individual items across time were all significant (*p*s < .001), and ranged from *r*s = .52 – .66.

The pattern of correlations between the 6-item SOAS and all other measures were similar to correlations reported with the 13-item SOAS (see Supplementary Table 3).

**Supplementary Table 2**

*Confirmatory Factor Analysis of the 6-Item SOAS in Study 2*

|  | Factor Loading (*SE*) |
| --- | --- |
| 5. I feel that this object has likes and dislikes. | .92 (.02) |
| 7. I feel that this object needs friends. | .79 (.04) |
| 9. I feel that this object knows what happens to it. | .93 (.02) |
| 10. I feel that this object can be excited or bored. | .94 (.02) |
| 11. I feel that this object can be scared or calm. | .93 (.02) |
| 12. I feel that this object knows right from wrong. | .94 (.02) |

*Note*. *N* = 420. All standardised factor loadings were significant (*p*s < .001).

**Supplementary Table 3**

*Means, Standard Deviations, and Spearman Correlations with the 6-Item SOAS in Study 2*

|  | *M* | *SD* | *r* |
| --- | --- | --- | --- |
| 6-Item SOAS | 1.51 | 2.77 | - |
| AMSR | 8.06 | 6.80 | .68^***^ |
| GATS | 27.91 | 18.06 | .75^***^ |
| Q-LES-Q-SF | 70.39 | 14.46 | -.04 |
| *Note*. *N* = 420. AMSR = Anthropomorphic Mental State Ratings, GATS = Graves Anthropomorphic Task Scale, SOAS = Specific Object Anthropomorphism Scale, Q-LES-Q-SF = Quality of Life, Enjoyment and Satisfaction Questionnaire – Short Form. *** *p* < .001. | | | |

**Specific Object Anthropomorphism Scale (Final)**

Think of an object. Write down what this object is: _________

Please tell us whether you agree with the sentences below. Answer based on how you feel right now.

Rating scale: 0 = “no” 1 = “maybe” 2 = “yes”

1. I feel that this object has likes and dislikes.

2. I feel that this object needs friends.

3. I feel that this object knows what happens to it.

4. I feel that this object can be excited or bored.

5. I feel that this object can be scared or calm.

6. I feel that this object knows right from wrong.
